# Sub-fertility in crossbred bulls: deciphering testicular level transcriptomic alterations between zebu (*Bos indicus*) and crossbred (*Bos taurus x Bos indicus*) bulls

**DOI:** 10.1186/s12864-020-06907-1

**Published:** 2020-07-21

**Authors:** Kamaraj Elango, Arumugam Kumaresan, Ankur Sharma, Pradeep Nag, Mani Arul Prakash, Manish Kumar Sinha, Ayyasamy Manimaran, Ebenezer Samuel King John Peter, Sakthivel Jeyakumar, Sellappan Selvaraju, Kerekoppa P. Ramesha, Tirtha K. Datta

**Affiliations:** 1 Theriogenology Laboratory, Veterinary Gynaecology and Obstetrics, Southern Regional Station of ICAR- National Dairy Research Institute, Bengaluru, Karnataka 560030 India; 2 Southern Regional Station of ICAR- National Dairy Research Institute, Bengaluru, Karnataka 560030 India; 3 Reproductive physiology Laboratory, ICAR - National Institute of Animal Nutrition and Physiology, Bengaluru, Karnataka 560030 India; 4 Animal Genomics Laboratory, ICAR - National Dairy Research Institute, Karnal, Haryana 132 001 India

**Keywords:** Crossbred, Sub-fertility, Zebu, Testis, Transcriptomics

## Abstract

**Background:**

The incidence of poor semen quality and sub-fertility/infertility is higher in crossbred as compared to Zebu males. Several attempts have been made to understand the possible reasons for higher incidence of fertility problems in crossbred males, at sperm phenotype, proteome and genome level but with variable results. Since the quality of the ejaculated spermatozoa is determined by the testicular environment, assessing the testicular transcriptome between these breeds would help in identifying the possible mechanisms associated with infertility in crossbred bulls. However, such information is not available. We performed global transcriptomic profiling of testicular tissue from crossbred and Zebu bulls using Agilent *Bos taurus* GXP 8X60k AMADID: 29411 array. To the best of our knowledge, this is the first study comparing the testicular mRNAs between crossbred and Zebu bulls.

**Results:**

Out of the 14,419 transcripts detected in bovine testis, 1466 were differentially expressed between crossbred and Zebu bulls, in which 1038 were upregulated and 428 were downregulated in crossbred bulls. *PI4KB* and *DPY19L2* genes, reported to be involved in sperm capacitation and acrosome formation respectively, were among the top 10 downregulated transcripts in crossbred testis. Genes involved in ubiquitination and proteolysis were upregulated, while genes involved in cell proliferation, stem cell differentiation, stem cell population maintenance, steroidogenesis, WNT signalling, protein localization to plasma membrane, endocannabinoid signalling, heparin binding, cAMP metabolism and GABA receptor activity were downregulated in crossbred testis. Among the 10 genes validated using qPCR, expression of *CCNYL, SOX2, MSMB, SPATA7, TNP1, TNP2* and *CRISP2* followed the same trend as observed in microarray analysis with *SPATA7* being significantly downregulated and transition proteins (*TNP1*, *TNP2*) being significantly upregulated in crossbred bulls.

**Conclusions:**

Abundant proteolysis by ubiquitination and downregulation of WNT signaling, cell proliferation, differentiation and steroidogenesis might be associated with higher incidence of poor semen quality and/or sub-fertility/infertility in crossbred bulls as compared to Zebu bulls. Downregulation of *SPATA7* (Spermatogenesis Associated 7) and upregulation of transition proteins (*TNP1* and *TNP2*) in crossbred bull testis might be associated with impaired spermatogenesis processes including improper chromatin compaction in crossbred bulls.

## Background

Although both males and females contribute to conception failure, infertility in a bull is formidable since a single male is used to artificially breed thousands of females [1]. It is well proved that the male offspring born out of species hybridization (for instance crossing of cattle with yak) are sterile. On the other hand, the male offspring produced by crossing *Bos taurus* males with *Bos indicus* females, tend to have higher incidence of sub-fertility/infertility problems as compared to both the parent breeds [2]. It is reported that, crossbred males are prone for many reproductive problems and possess higher culling rate of 40–70% due to sub-fertility and poor semen freezability [3, 4]. Moreover, ejaculate rejection rate (owing to poor semen quality) in crossbred bulls ranged from 10 to 100% with the average of 55% [5–9]. Ejaculates were rejected for one or more reasons that include low sperm concentration, poor mass activity, initial motility and poor progressive motility. Further, it was observed that, post-thaw sperm motility decreased while the exotic inheritance of the crossbred bulls increased [10]. In the world of Zebu bulls, *albeit* libido is poor, infertility is less when compared to crossbred bulls [7, 11].

Though axiomatic evidences of inferior reproductive performances in crossbred bulls than Zebu bulls has been demonstrated in terms of genetic [10], hormonal [12], semenological [7] and andrological aspects [13], the etiology has not been well understood. Our group has been working towards the goal of unravelling the reasons for infertility in crossbred bulls, and found the differences in terms of proportion of sertoli cells in relation to spermatogenic cells [13], proteomic profile of spermatozoa [14], seminal plasma [15], and spermatogenic and sertoli cells [16]. A majority of these studies indicate altered sperm quality and functions, as the major factor contributing to high incidence of infertility in crossbred bulls. Several studies indicate the relationship of sperm transcripts with sperm function and fertility [17–20]. The development of spermatogonia into mature spermatozoa requires a lengthy duration and a series of complex physiological changes at testicular level. Spermatozoa carry thousands of different types of RNA from the testis [21–24]. Spermatozoal transcripts are having their putative purpose in spermatogenesis [25], sperm function [26], fertilization [27] and embryo development [28]. Number of mRNA found in the spermatozoa are only 50% of the mRNA found in the testis of men [29] and stallion [30]. The testis specific transcripts such as CatSper (Cation channels of sperm) has been reported to be involved in calcium influx into the sperm tail resulting in hyperactivated motility and capacitation [31]. Further, few earlier studies assessed the dynamics of transcripts in sperm having different motilities [32], after meiosis [33], after capacitation [34] and after cryopreservation [35] and indicated their role in sperm functions. It has been reported that compatibility between two gametes is essential for the consolidation process (transfer of RNA based information to a chromatized state), which will be impaired when crossing is carried out between species or between breeds [36]. Moreover, it causes dissimilarities between pseudoautosomal regions (a short identical sequence exists between X and Y chromosomes) of sex chromosomes, which results in impaired sex chromosome pairing and segregation during meiosis [37–39]. In this line, breed variations in testicular transcriptome and associated sexual function development has been reported [40, 41]. While a plethora of reports are available about spermatozoal transcripts, information on transcriptomic profile of testis, an important reproductive and endocrine organ critical for spermatogenesis and sperm quality, is very limited. Therefore, we envisaged that studying the testicular transcripts between Zebu and crossbred cattle would help us to understand aetiology of infertility in the later breed.

In the present study, we performed transcriptomic profiling of testicular tissue collected from crossbred and indigenous bulls using Agilent *Bos taurus* GXP 8X60k AMADID: 29411 Chip. Our objective was to identify the molecular signatures and their pathways associated with the higher incidence of subfertility in crossbred bulls as compared to Zebu breed.

## Results

### Detection of global mRNA in bovine testis using microarray

Microarray experiment was performed to identify the differences in transcripts between crossbred and Zebu bull testis using bovine microarray chip (AMADID: 29411) which encompassed 51,282 probes for 32,429 genes. A total of 14,419 transcripts were detected in the current study after removal of duplicate transcripts and transcripts with compromised signals. The 10 highly expressed transcripts in bovine testes are given in Additional file 1. Unexpectedly, pregnancy associated glycoproteins, placenta specific transcripts and placenta related transcripts (Table 1) were also detected in bovine testis.

**Table 1:** List of pregnancy associated and placenta related genes expressed in cattle testes

Gene symbol	Gene name	Genbank accession
PAG11	Pregnancy-associated glycoprotein 11	NM_176623
PAG12	Pregnancy-associated glycoprotein 12	NM_176622
PAG7	Pregnancy-associated glycoprotein 7	NM_176618
PAG5	Pregnancy-associated glycoprotein 5	NM_176616
PAG4	Pregnancy-associated glycoprotein 4	NM_176615
PAG16	Pregnancy-associated glycoprotein 16	NM_176625
PAG19	Pregnancy-associated glycoprotein 19	NM_176628
MGC157405	Pregnancy-associated glycoprotein	NM_001083697
LOC784242	Pregnancy-associated glycoprotein 18-like	
MGC157408	Pregnancy-associated glycoprotein	NM_001105381
PLAC1	Placenta-specific 1	NM_001077057
PLAC9	Placenta-specific 9	NM_001038521
PLAC8	Placenta-specific 8	NM_001025325
TEPP	Testis, prostate and placenta expressed	BC110152
CDH3	Cadherin 3, type 1, P-cadherin (placental)	XM_614683
PGF	Placental growth factor	NM_173950
20ALPHA-HSD	Placental and ovary 20alpha hydroxysteroid dehydrogenase protein	NM_001167660

### Functional classification and pathway analysis of transcripts detected in bovine testis

Gene ontology analysis and pathway enrichment of bovine testicular transcripts (14419) was performed using Panther (Version 14.1) database. Gene ontology analysis revealed involvement of 13,983 genes in biological process, 9756 genes in cellular component and 10,338 genes in molecular function, while 665 genes were unannotated. Among the 13,983 genes involved in biological process, a majority of them were involved in cellular process (4430), metabolic process (3197) and biological regulation (2075). Also, some of the genes were involved in localization (1541), immune system process (238) and reproduction (161). Among the 9756 genes involved in cellular component, a majority of them were related to cell (4210), organelle (2947), protein containing complex (1148) and membrane (760). Among the 10,338 genes involved in molecular function, 3675, 3637 and 711 genes were involved in binding, catalytic and transporter activity, respectively. Pathway enrichment of testicular transcripts revealed 160 different pathways. Among these pathways, 216, 209 and 115 number of genes were detected to be involved in Gonadotropin-releasing hormone receptor pathway, WNT signalling pathway and EGF receptor signalling pathway, respectively.

### Differences in transcriptional abundance of genes between crossbred and zebu testis

Among 14,419 detected transcripts, 1466 were differentially expressed between crossbred and Zebu bulls, in which 1038 transcripts were upregulated (≥1.5 fold change) and 428 transcripts were downregulated (≤ − 1.5 fold change) in crossbred testis as compared to Zebu testis. Among the differentially expressed transcripts, 10 highly upregulated and downregulated transcripts are shown in Table 2.

**Table 2:** Top 10 upregulated and downregulated transcripts in crossbred as compared to indigenous bull testes

Gene symbol	Gene name	Fold change
**Upregulated**		
XKRX	XK, Kell blood group complex subunit-related, X-linked	**9.68**
ARR3	Arrestin 3, retinal (X-arrestin)	**8.84**
C7H5orf46	Hypothetical protein LOC614113	**8.31**
OR52K1	Olfactory receptor, family 52, subfamily K, member 1	**8.02**
LOC100140710	Similar to fel d I chain 1 precursor with leader B	**8.02**
OR12D2	Olfactory receptor, family 12, subfamily D, member 2	**7.81**
LOC783978	Family with sequence similarity 126, member A	**7.45**
LOC788626	Similar to hCG1811267	**7.14**
IL12RB2	Interleukin 12 receptor, beta 2	**6.84**
MAP 6	Microtubule-associated protein 6	**6.76**
**Downregulated**		
C10H5orf13	Neuronal protein 3.1	**−10.05**
PI4KB	Phosphatidylinositol 4-kinase, catalytic, beta	**−9.20**
CTDSP2	CTD (carboxy-terminal domain, RNA polymerase II, polypeptide A) small phosphatase 2	**−9.04**
RDH11	Retinol dehydrogenase 11 (all-trans/9-cis/11-cis)	**−8.54**
RNF128	Ring finger protein 128	**−7.86**
CCDC12	Coiled-coil domain containing 12	**−7.71**
DAPL1	Death associated protein-like 1	**−7.58**
DPY19L2	Dpy-19-like 2 (*C. elegans*)	**−7.58**
FASTKD1	FAST kinase domains 1	**−7.45**
TTYH1	Tweety homolog 1 (Drosophila)	**−6.81**

### Functional classification of upregulated transcripts in the crossbred bulls

**Fig. 1:**
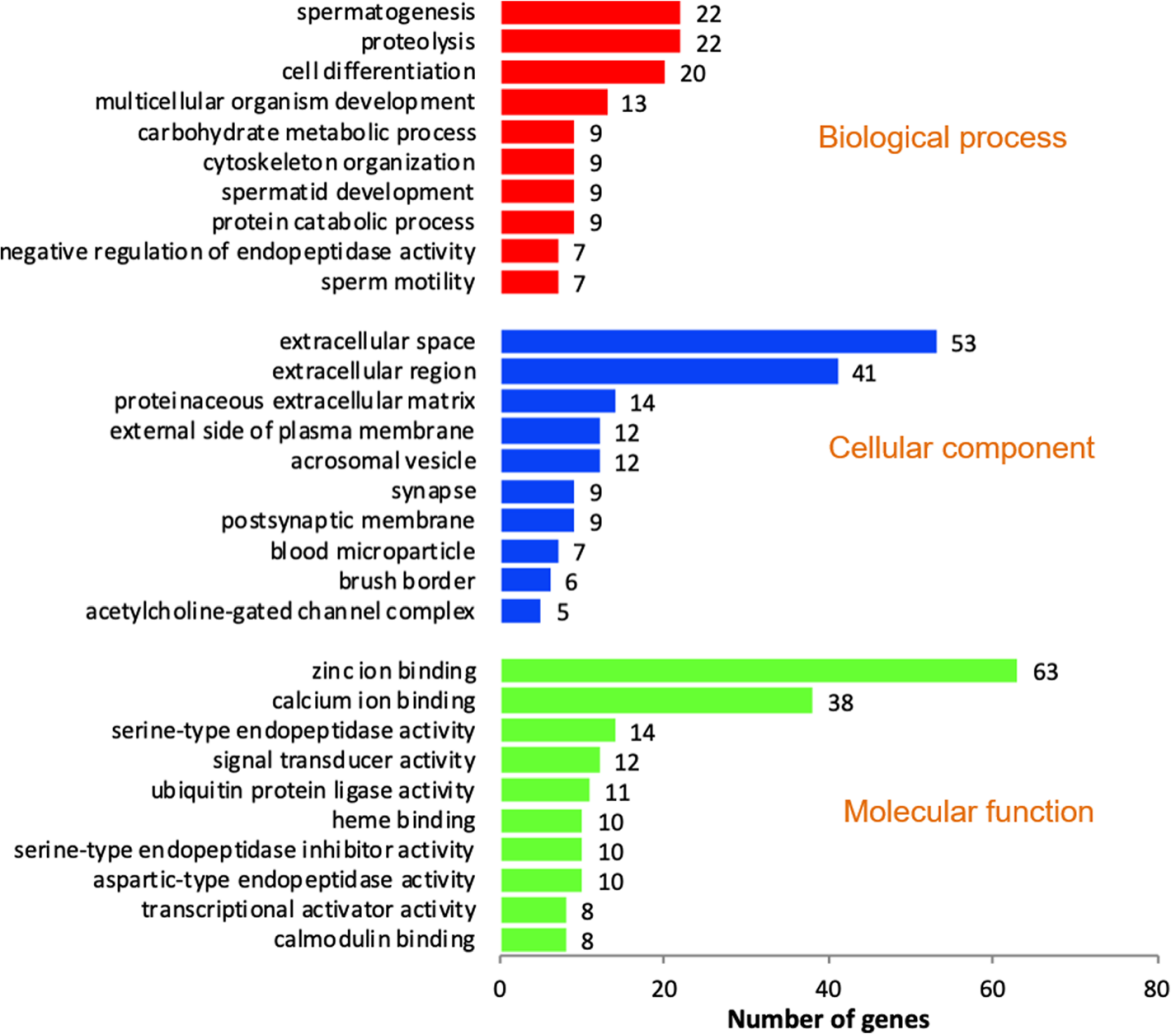
Functional classification of upregulated transcripts in crossbred testes based on the gene ontology terms

**Fig. 2:**
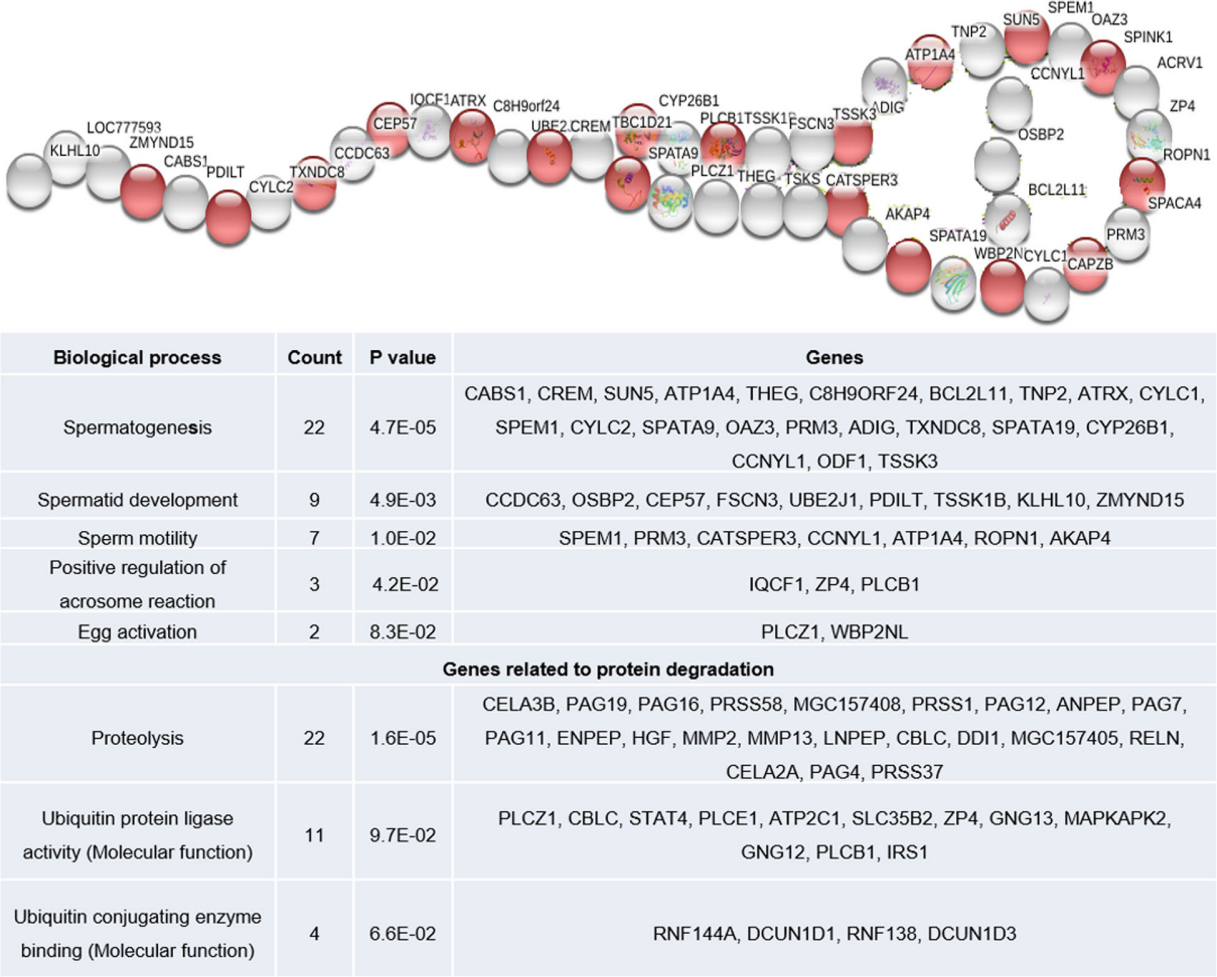
Spermatogenesis (red) and sperm function (white) related up regulated genes in crossbred testis

Ontology analysis of upregulated genes revealed their involvement in 38 biological process, 12 cellular components and 22 molecular functions. Among this, top 10 biological processes, cellular components and molecular functions are shown in Fig. 1. Among the cellular components observed, “acrosomal vesicle” was found to be specific to spermatozoa, in which 12 genes (*SPACA4*: Sperm acrosome associated4; *LOC777593, TSKS*: Testis-specific Serine Kinase Substrate; *IQCF1*: IQ motif containing F1; *CATSPER3*: Cation channel, sperm associated 3; *TXNDC8*: Thioredoxin domain containing 8; *ZP4*: Zona pellucida glycoprotein 4; *SPINK1*: Serine peptidase inhibitor, Kazal type 1; *ACRV1*: Acrosomal vesicle protein 1; *TSSK1B*: Testis-specific serine kinase 1B; *TBC1D21*: TBC1 domain family and *CAPZB*: Capping protein (actin filament) muscle Z-line, beta) were detected. Among these 12 genes, *SPINK1* (Serine peptidase inhibitor, Kazal type 1) was highly upregulated. Genes with molecular function such as calcium ion binding (38 genes), ubiquitin protein ligase activity (11 genes) and ubiquitin conjugating enzyme binding (4 genes) were upregulated in crossbred bulls. A total of 15 ubiquitination related genes have been upregulated. Genes involved in spermatogenesis and sperm function related biological process such as spermatogenesis, spermatid development, sperm motility, egg activation and positive regulation of acrosome reaction were also upregulated in crossbred testis. Upregulated spermatogenesis and sperm function related genes are shown in Fig. 2 (complete genes names are given in Additional file 10) and interaction between these genes are shown in Additional file 2. Among upregulated biological process, proteolysis is the highly significant (*P* = 1.60E-05) biological process and it was observed that, 22 genes involved in proteolysis were upregulated in crossbred testis (Fig. 2), which includes serine proteases, chymotrypsin like elastases and matrix metallopeptidases.

### Functional classification of downregulated transcripts in the crossbred bulls

**Fig. 3:**
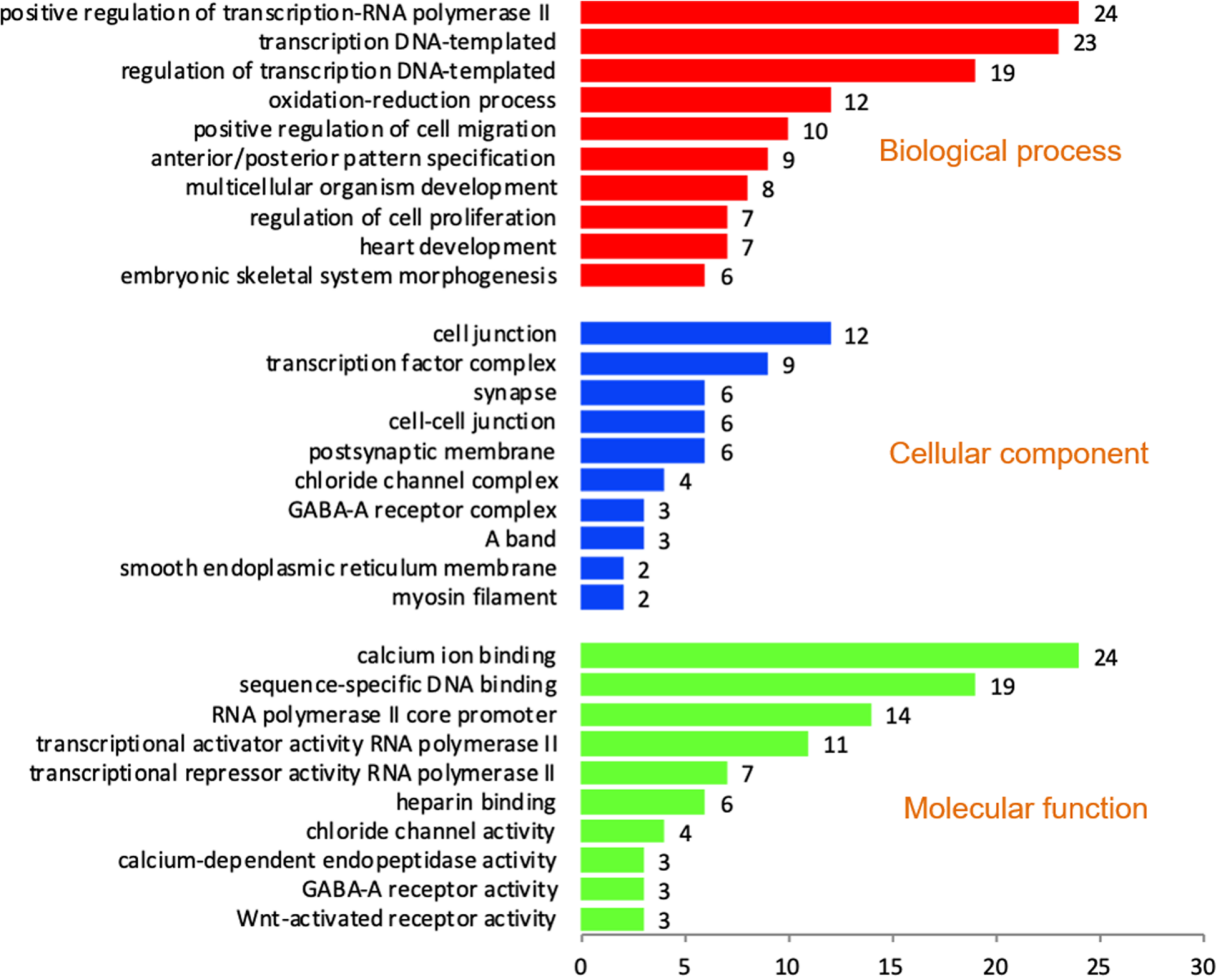
Functional classification of downregulated transcripts in the crossbred bulls based on the gene ontology terms

**Fig. 4:**
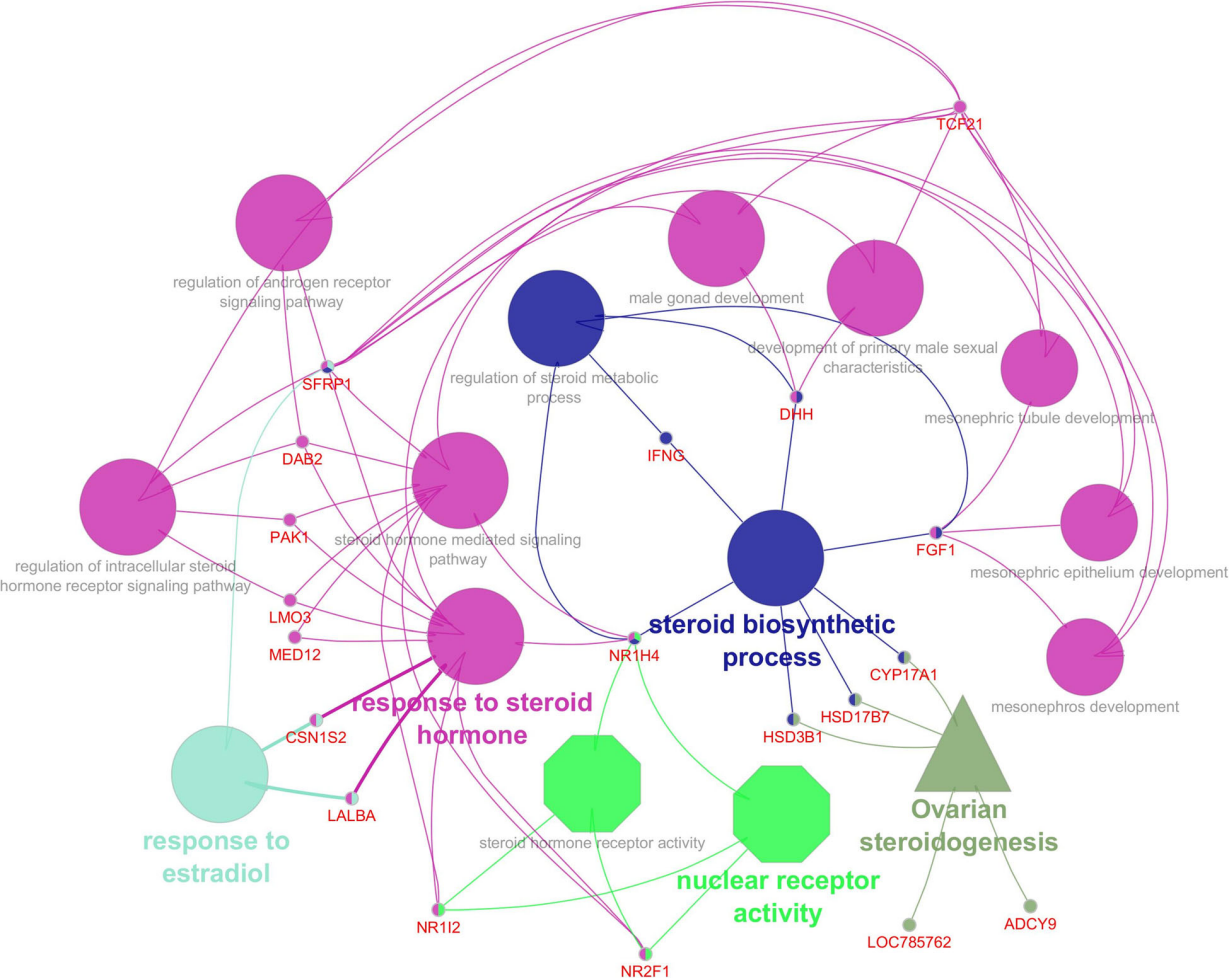
Downregulated steroidogenesis related genes in crossbred testis and their biological process (Elliptical), molecular functions (octagonal) and pathway (triangle)

Ontology analysis revealed the involvement of downregulated genes in 58 biological processes, 10 cellular components and 13 molecular functions. Among this, top 10 in each process are shown in Fig. 3. We observed that, downregulated genes were involved in biological process such as cell proliferation, stem cell differentiation, stem cell population maintenance, establishment of protein localization to plasma membrane, male gonad development, fertilization, cAMP metabolic process, WNT signalling and steroidogenesis (Additional file 3). Genes involved in molecular function such as heparin binding and WNT activated receptor activity were also downregulated. List of downregulated genes in crossbred males related to spermatogenesis and sperm function are shown in Additional file 4. Totally 21 genes (*CYP17A1*: Cytochrome P450 Family 17 Subfamily A Member 1; *DAB2*: Disabled homolog 2; *DHH*: Desert hedgehog; *FGF1*: Fibroblast Growth Factor 1; *GSTA3*: Glutathione S-transferase alpha 3; *HSD17B7*: 17 Beta-Hydroxysteroid Dehydrogenase; *HSD3B1*: 3-beta-hydroxysteroid dehydrogenase; *IFNG*: Interferon gamma; *LALBA*: Lactalbumin Alpha; *LMO3*: LIM Domain Only; *MED12*: Mediator Complex Subunit 1; *NR1H4*: Nuclear Receptor Subfamily 1 Group H Member 4; *NR1I2*: Nuclear Receptor Subfamily 1 Group I Member 2; *NR2F1*: Nuclear Receptor Subfamily 2 Group F Member 1; *PAK1*: Serine/threonine-protein kinase; *SFRP1*: Secreted Frizzled Related Protein 1; *TCF21*: Transcription factor 21; *ADCY9*: Adenylate Cyclase 9 and *CSN1S2*: Casein alpha-S2; *LOC508455* and *LOC785762*) involved in steroidogenesis (Based on Cluego app v 2.5.8 results) were downregulated in crossbred bulls. Interaction among these genes are shown in Fig. 4. In spite of the up regulation of few genes involved in WNT signalling (WNT2, WNT3, WIF1), majority of genes were downregulated (*ATP6V0C*: ATPase H+ Transporting V0 Subunit C; *DAB2*: Disabled homolog 2; *EDA*: Ectodysplasin A; *FZD4*: Frizzled Class Receptor 4; *GSC*: Goosecoid; *HHEX*: Hematopoietically-expressed homeobox; *MED12*: Mediator Complex Subunit 12; *NKD2*: Naked cuticle 2; *NOTCH1*: Notch homolog 1; *ROR2*: Receptor Tyrosine Kinase Like Orphan Receptor 2; *RSPO3*: R-Spondin 3; *SFRP1*: Secreted Frizzled Related Protein 1; *SOSTDC1*: Sclerostin domain containing 1; *TMEM198*: Transmembrane protein 198 and *ZEB2*: Zinc finger E-box-binding homeobox 2). Among the three upregulated genes involved in WNT signalling, WNT inhibitory factor 1 (*WIF1*) was highly upregulated (Additional file 5).

### Pathway analysis of differentially expressed transcripts in crossbred bull testis

Pathway analysis by DAVID bioinformatics tool (V 6.8, Laboratory of Human Retrovirology and Immunoinformatics) revealed the involvement of genes in 16 different upregulated pathways (Top 10 pathways are shown in Additional file 6). Among these upregulated pathways, PI3K-Akt signalling pathway (bta04151) and Jak-STAT signalling pathway (bta04630) are related to spermatogenesis and sperm functions. A total of 21 genes are involved in PI3K-Akt signalling pathway and 13 genes are involved in Jak-STAT signalling pathway (Additional file 7). Among the 10 downregulated pathways, 5 genes are involved in each retrograde endocannabinoid signalling (bta04723), GABAergic synapse (bta04727) and other four genes are involved in ovarian steroidogenesis (bta04913).

### Real time expression analysis of select genes

**Fig. 5:**
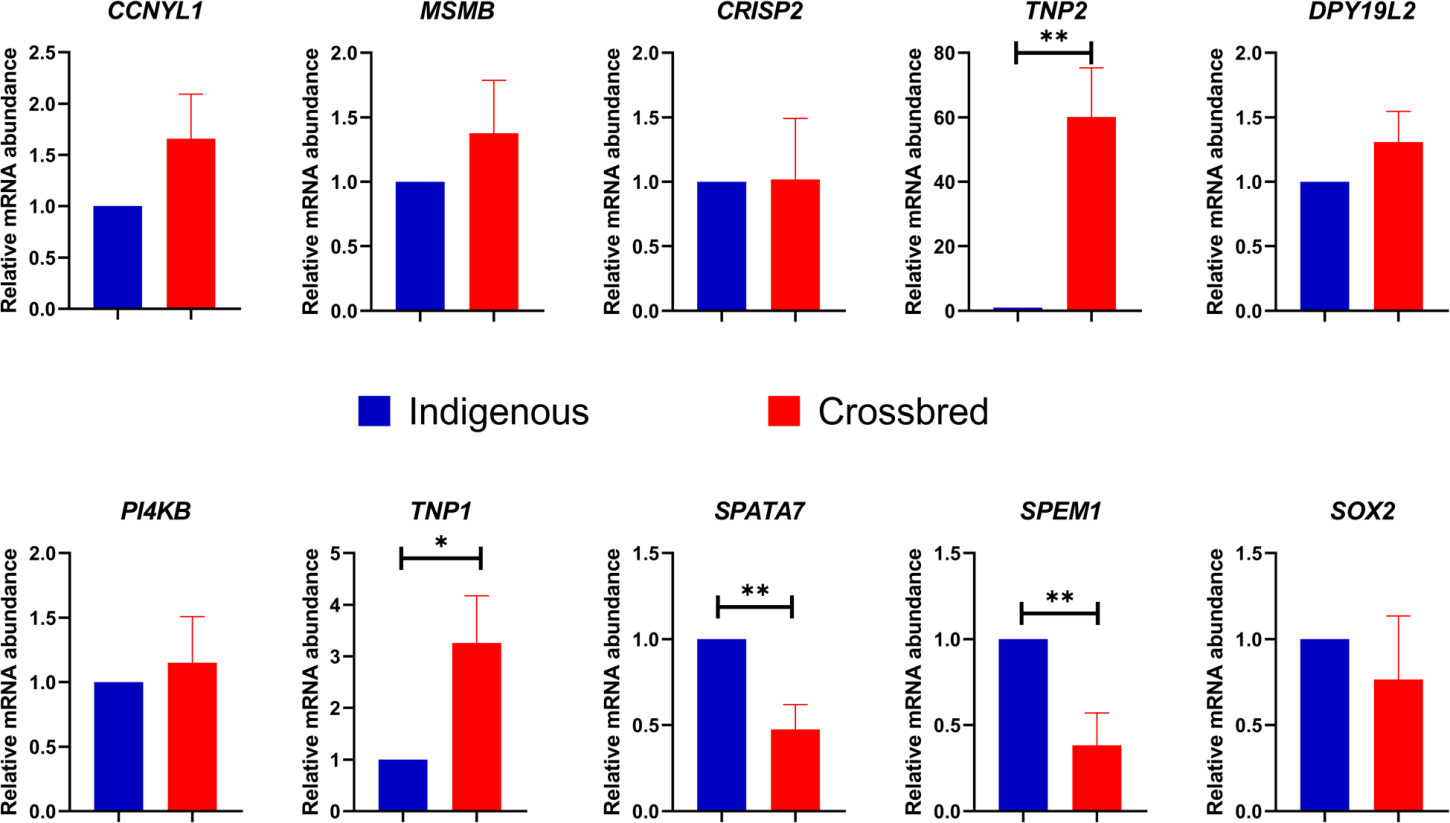
Relative expression of selected genes in crossbred and indigenous testes (* denotes *p* < 0.05 and ** denotes *p* < 0.01)

Ten genes (*MSMB*: Microseminoprotein Beta; *CCNYL-1*: Cyclin Y like 1; *PI4KB*: Phosphatidylinositol 4-kinase beta; *DPY19L2*: DPY19 like 2; *SPATA7*: Spermatogenesis associated 7; *CRISP2*: Cysteine-rich secretory protein 2; *TNP1*: Transition protein 1; *TNP2*: Transition protein 2; *SPEM1*: Spermatid maturation 1 and *SOX2*: SRY-box 2) were selected for qPCR expression analysis based on fold change in microarray and their role in spermatogenesis and sperm functions (Additional file 8). Results of qPCR expression analysis are shown in Fig. 5. Among the 10 genes validated using qPCR, expression of *CCNYL*, *MSMB, SOX2, SPATA7, TNP1, TNP2* and *CRISP2* followed the same trend as observed in microarray analysis. Among the 7 genes following same trend, *SPATA7* was significantly downregulated, whereas *TNP1* and *TNP2* were significantly upregulated in crossbred males. On the other hand, expression of *DPY19L2*, *PI4KB* and *SPEM1* followed the opposite trend as compared to microarray, in which the latter is significantly upregulated in crossbred males.

## Discussion

To understand the possible reasons for higher incidence of sub-fertility in crossbred bulls, we performed global transcriptomic profiling of testicular tissue from crossbred and Zebu bulls. A total of 14,419 transcripts were detected in bovine testis. The presence of pregnancy associated glycoproteins, placenta specific and related transcripts in bovine testis in our study, suggesting the role of spermatozoa beyond fertilization and embryonic development. The list of 10 highly upregulated and downregulated transcripts in crossbred testis is shown in Table 2. *IL12RB2*: Interleukin 12 Receptor Subunit Beta 2, a gene reported to be associated sperm plasma membrane damage by producing interferon-gamma [42], was one among the top 10 upregulated transcripts in crossbred testis. *PI4KB* and *DPY19L2* genes, reported to be involved in actin polymerization [43] and acrosome formation [44], respectively, were among the top 10 downregulated transcripts in crossbred testis.

### Functional annotation of differentially expressed transcripts in the crossbred testis

#### Poor protein stability

Functional annotation of differentially expressed transcripts revealed that 22 genes involved in proteolysis and 15 genes involved in ubiquitination were upregulated in crossbred testis. Ubiquitination is the terminal stage of apoptotic processes in testis and involved in protein degradation. It is negatively correlated with seminal parameters such as concentration (suggests sperm degradation in epididymis), motility, morphology and chromatin integrity (suggests apoptosis) [45, 46]. Genes involved in protein localization to plasma membrane and majority of the genes involved in WNT pathway were also downregulated in crossbred bulls. WNT pathway initiates sperm motility and maintains protein stability by inhibiting poly-ubiquitination of proteins [47, 48]. Down regulation of genes involved in WNT pathway (inhibitor of protein degradation), protein localization to plasma membrane and upregulation of genes involved in ubiquitination and proteolysis are clearly indicating that inability of the crossbred bull testis to maintain protein integrity could be the major contributing factor for sub-fertility/infertility in crossbred bulls.

#### Perturbed steroidogenesis

Downregulation of steroidogenesis related genes in crossbred bull testis is an exciting finding in this study. Downregulated *CYP17A1* gene is involved in the regulation of CYP17A1 enzyme that possess 17 α-hydroxylase activity and 17, 20-lyase activity, which are crucial for steroidogenesis [49]. Testosterone plays a role in testicular descent, sexual differentiation, sexual characters development [50] and spermiogenesis [51]. Gulia et al*,* found that testosterone is significantly lower in crossbred bulls than those of Zebu bulls [12]. Progesterone is important for sperm motility, capacitation, hyperactivation, acrosome reaction and directing the sperm towards egg [52, 53]. Progesterone mediated modulation in sperm function is happening through the Gama amino butyric acid (GABA_A_) receptors present on the sperm plasma membrane [54, 55]. In our study, genes involved in GABAergic synapse pathway, which is involved GABA synthesis is also downregulated in crossbred testis. These results are indicating the alteration of steroidogenesis in crossbred bulls could be the reason for infertility.

#### Altered spermatogenesis and sperm function

Downregulation of genes involved in cell proliferation, stem cell differentiation, stem cell population maintenance may have negative effect on cell differentiation during spermatogenesis. Six genes involved in heparin binding are downregulated in crossbred bulls. Heparin binding proteins are involved in capacitation and it could be the biomarker of male fertility [56, 57]. cAMP and calcium ion play a prime role for the sperm to undergo capacitation and acrosome reaction [58–61]. In our study, 24 genes involved in calcium ion binding and 2 genes involved in cAMP metabolism were downregulated in crossbred testis. This indicates the possibilities of compromised capacitation and acrosome reaction in crossbred bulls. Consistent with our earlier results, pre-mature capacitation is a significant problem in crossbred bulls [14]. However, 38 genes involved in calcium ion binding were also upregulated. Therefore, genes involved in calcium ion binding need to be studied further for understanding their roles in crossbred testis. Genes involved in spermatogenesis, spermatid development, sperm motility, egg activation and positive regulation of acrosome reaction were upregulated in crossbred testis. Among these genes, some are having positive roles (*PRM3*: Protamine 3 is vital for sperm chromatin integrity) [62, 63], some others are having negative roles (*ELSPBP1*: Epididymal Sperm Binding Protein 1- Marker of dead sperm and found at higher level in sub-fertile bull’s semen) [64, 65], while few others are reported to have both positive and negative roles on spermatogenesis and sperm function (*SPEM1*: Spermatid maturation protein 1*-* essential for spermatid maturation, but its involvement in the ubiquitination is also reported) [66]. However, roles of these transcripts in bull fertility still remains inconclusive.

### Pathway analysis of upregulated and downregulated transcripts in crossbred bull testis

Among the upregulated pathways, PI3K-Akt signalling pathway and Jak-STAT signalling pathway are important for capacitation and acrosome reaction [67–71]. Even though these pathways are reportedly having positive roles in sperm function, transcripts detected in this pathway in our study have negative roles in spermatogenesis and sperm function viz. *IRS1*: Insulin Receptor Substrate 1 (Involved in ubiquitination) [72], *BCL2L11* (apoptotic-facilitating gene) [73], *PRL*: Prolactin (higher seminal prolactin is decreasing the sperm reproductive capacity) [74], *IL12RB2*: Interleukin 12 Receptor Subunit Beta 2 (damaging effect on sperm membrane integrity) [42] and *LEP*: Leptin (seminal leptin is negatively correlated with progressive motility of sperm and testosterone concentration in serum) [75]. Among the downregulated pathways, endocannabinoid signalling pathway, steroidogenesis and GABAergic synapse are related to the spermatogenesis and sperm function. Endocannabinoid system is involved in bovine sperm-oviduct binding, capacitation, release of sperm from oviductal epithelium and it influences sperm capacitation by heparin signalling pathway [76–78]. In our study, both endocannabinoid signalling and heparin binding related genes were downregulated. It indicates the possible alterations in capacitation process of crossbred bull spermatozoa.

### Real time expression analysis of selected genes

To validate the microarray data, we selected ten genes for further validation by RT-qPCR. Even though, *CCNYL1* (regulator of WNT signalling, sperm motility and spermatogenesis) [48, 79], *CRISP2* (involved in sperm egg interaction) [80] and *MSMB* (inhibitor of Na^+^–K^+^–ATPase activity, sperm motility and acrosome reaction) [81–83] were upregulated in crossbred males in microarray, however, no significant difference was observed between crossbred and Zebu bulls in qPCR expression analysis. *PI4KB* (involved in actin formation) [43, 84], *SOX2* (essential for early embryonic development) [85] and *DPY19L2* (causes infertility by impairing head elongation, PLCζ signalling, acrosome formation and by causing globospermia) [44, 86] were downregulated in crossbred males, in microarray. However, in qPCR expression analysis, no significant difference is observed between crossbred and Zebu bulls. Unexpectedly, *SPEM1* (involved in ubiquitination during spermatogenesis along with *UBQLN1*) [66], which is upregulated in microarray, was significantly downregulated in qPCR. *SPATA7* plays a role in initiating meiotic recombination by preparing chromatin [87] and act as a candidate gene for sperm motility [88]. In the final stage of spermatogenesis, histones should be replaced by transition proteins (TNPs) and then TNPs should be finally replaced by protamines to facilitate chromatin compaction [89]. According to our data from both microarray and qPCR analysis, *SPATA7* is downregulated (indicates alteration of spermatogenesis) and transition proteins (*TNP1*, *TNP2*) were upregulated (indicates the retention of TNPs, improper protamination and chromatin condensation) in crossbred males as compared to Zebu males.

## Conclusions

Abundant proteolysis by ubiquitination and downregulation of WNT signalling, cell proliferation, differentiation and steroidogenesis might be the reason for higher incidence of poor semen quality and/or sub-fertility/infertility in crossbred bulls as compared to Zebu bulls. Downregulation of *SPATA7* (Spermatogenesis Associated 7) and upregulation of transition proteins (*TNP1* and *TNP2*) in crossbred bull testis might be associated with impaired spermatogenesis including chromatin compaction.

## Methods

### Experimental animals and sample collection

The experimental procedure was duly approved by the Institute Animal Ethics Committee (CPCSEA/IAEC/LA/SRS-ICAR-NDRI-2019/No.18) and performed in accordance with relevant guidelines and regulations. Three crossbred (Holstein Friesian crossbred with 50–75% exotic inheritance) and three Zebu (Tharparkar breed) bulls aged 24 months were sampled from Livestock Research Complex, ICAR - National Dairy Research Institute, Karnal. Testis of each animal was obtained by unilateral castration. Bulls were restrained at lateral recumbency and sedated with Xylazine hydrochloride (Xylaxin, Indian Immunologicals, India) at the dose rate of 0.1 mg/kg body weight. After sedation, the site was shaved, tincture iodine was applied to the site and cleaned thoroughly to ensure asepsis. The site of incision was infiltrated with 6–8 ml of 2% lignocaine (Cadila Health care Ltd., India) at the level of the spermatic cord. The lower part of scrotum was incised with the help of surgical scalpel (B.P. blade no. 23). Right testis of each animal was exposed, and the spermatic cord was ligated tightly using catgut (Size3–0; Stericat Gutstrings (P) Ltd., India). After ligation intact testicle was removed; fat and fascia surrounding the testis were resected and two crosscut slices of the testicular tissues in the middle of testis were obtained by fine scale dissection. All crosscut slices were placed in individual sterile containers containing normal saline with penicillin streptomycin (Sigma Aldrich, USA), transported to laboratory and stored at − 80 °C until RNA isolation. All the bulls were given due post-operative care as per standard veterinary protocol. After the study, the bulls were retained in the farm and used as teasers.

### Microarray analysis

#### RNA extraction

Total RNA was isolated from crossbred and Zebu bull testis using Qiagen RNeasy Mini Kit as per the manufacturer’s instructions. Bioanalyzer was used to assess the total RNA quality control and samples with optimal purity (OD 260/280 > 1.9 and < 2.0 and optimal concentration (> 100 ng/mL) were selected for further use in microarray. Group wise pooling of RNA in equal quantity (Three samples from crossbred group and three samples from Zebu group) was done for microarray analysis.

#### Labelling protocol

Oligonucleotide probe sequences were used to develop Agilent *Bos taurus* GXP 8X60k AMADID: 29411 slide by Agilent Technologies. Agilent’s Quick-Amp labeling Kit (part number = 5190–0442) was used for labelling the samples. T7 promoter based-linear amplification method was used for generating labelled complementary RNA (One-Color Microarray-Based Gene Expression Analysis). Pooled RNA from each group was reverse transcribed using oligo dT based method. mRNA was primed with oligo dT primer tagged to T7 promoter sequence and converted into double stranded cDNA. Further the cDNA was converted to cRNA by in-vitro transcription reaction by T7 RNA polymerase enzyme in presence of Cy3 dye. During this cRNA synthesis, Cy3 labelled Cytosine nucleotide was incorporated into the newly synthesized strands. Labelled cRNA thus obtained was cleaned up using Qiagen RNeasy columns (Qiagen, Cat No: 74106). The concentration and the amount of dye incorporated in the cRNA were assessed using NanoDrop ND-1000.

#### Hybridization protocol

Specific activities of the labelled samples were determined. The QC passed samples (specific activity > 10) were proceeded for hybridization. Labelled cRNA were fragmented and hybridized on the bovine array (AMADID: 29411), which encompasses a total of 51,282 bovine probes for 32,429 genes using Agilent’s In situ Hybridzation kit (5188–5242) in Sure Hybridization Chambers (Agilent) at 65 °C for 16 h.

#### Scan and data processing

Microarray slides were scanned using the Agilent Microarray Scanner (Agilent Technologies, Part Number G2600D). Feature Extraction Software (Version-11.5, Agilent) was used to quantify the images. Extracted raw data were analyzed using GeneSpring GX software from Agilent. The normalization was performed using GeneSpring GX 12.6 Software using 75th percentile shift. In this normalization, gProcessed signal (dye normalized background subtracted signal intensity) is log transformed and then for each of the array the 75th percentile value is calculated separately. In each sample the log transformed intensity values for each probe is subtracted by the calculated 75th percentile value of the respective array and expression values are obtained. Differentially expressed genes were identified by calculating fold change of expression values (log base2) with respect to control samples. Differentially expressed genes include upregulated (> 1.5 fold) and downregulated genes (< − 1.5 fold). The list of differentially expressed transcripts between Zebu and crossbred bull testis are given in Additional file 9.

### Annotation of genes detected in microarray

Annotation of genes carried out by online bioinformatic resources based on the existing information about *Bos taurus* genes. Gene ontology (GO) analysis and pathway analysis of differentially expressed transcripts were carried out using DAVID Bioinformatics Resources 6.8 (Laboratory of Human Retrovirology and Immunoinformatics, USA) based on Huang et al. protocols [90]. However, gene ontology analysis of overall expressed transcripts was done by PANTHER (Version 14.1) based on Mi et al. protocols [91]. Interactions between genes possessing functions and pathways related to spermatogenesis and sperm function were analyzed using Cluego app (v2.5.3, Integrative Cancer Immunology, Jerome Galon) via Cytoscape bio informatics software platform [3.7.1, U.S. National Institute of General Medical Sciences (NIGMS), USA].

### Validation of microarray results

Validation of microarray results was carried out using testes samples from 10 crossbred males and 10 Zebu males. Fat and fascia surrounding the testis were resected and two crosscut slices of the testicular tissues in the middle of testis were obtained by fine scale dissection. All crosscut slices were snap frozen in liquid nitrogen (− 196 °C), transported to laboratory and stored until RNA isolation. Total RNA was isolated from these samples using PureLink RNA Mini Kit (Thermo-scientific, USA) according to the manufacturer’s instructions. The isolated RNA was subjected to DNase treatment using TURBO DNA- *free* Kit (Thermo Fisher Scientific). RNA quantification and quality assessment were determined using Nanodrop (ND-1000, Thermo-scientific, USA). RNA samples with 260/280 ratio of 1.9 to 2.0 were processed for cDNA synthesis. cDNA was synthesized using RevertAid First Strand cDNA Synthesis Kit (Thermo-scientific, USA) by keeping the initial concentration of 1000 ng of total RNA per reaction.

#### Real time quantitative polymerase chain reaction (RT-qPCR)

The expression of 10 differentially expressed genes, which were selected based on fold change and their role in spermatogenesis and sperm function (Additional file 8) was verified by qPCR. Primers were designed using PRIMER-3 across exon-exon junctions in order to eliminate the contaminating genomic DNA amplification and procured from Integrated DNA Technologies, USA. The annealing temperatures of primers for the selected genes were optimized using PCR and the cDNA prepared from different samples were subjected to qPCR analysis. Expressions of traditional housekeeping genes (*GAPDH* and *BACTIN*) were studied in ten crossbred and ten Zebu testis samples. *GAPDH* was stable based on the method described by Schmittgen & Livak [92]. Real-time quantitative PCR was performed on Insta Q96 Plus Real Time Machine PCR system (HiMedia, India) in a 15 μL reaction comprising 1 μL cDNA, 0.25 μL (10 pmol/ μL) forward and reverse primers, and 7.5 μL of Maxima SYBR Green/ROX qPCR master mix 2X. The thermal cycling conditions consisted of initial denaturation at 95 °C for 10 min, followed by 40 cycles of 95 °C for 15 s, 60 °C for 30 s, and 72 °C for 30 s. Relative gene expression levels were determined using the 2 ^-∆∆Ct^ method [92], where ∆Ct = Ct _target_ – Ct _internal reference_ and ∆∆Ct = ∆Ct _target_ - ∆Ct _calibrator_. *GAPDH* served as the internal reference gene. Gene expression data were normalised against *GAPDH* expression. The calibrator in each study consisted of cDNA from the corresponding control group. Relative mRNA expression is expressed as n-fold mRNA expression relative to the calibrator. The differences in fold change values between two groups were evaluated by t-Test using SPSS (22.0, IBM, USA) software. The difference was considered as significant when *P* < 0.05. The specificity and integrity of the PCR products were ensured by melting curve analysis, whereas the appropriateness of size was confirmed by agarose gel electrophoresis. The experiment was repeated thrice, each time in duplicates.

## Supplementary information

**Additional file 1.** Top 10 abundant transcripts in bovine testis

**Additional file 2.** Share of biological process between upregulated genes related to spermatogenesis and sperm function in crossbred testis

**Additional file 3.** Share of biological process between downregulated genes related to spermatogenesis and sperm function in crossbred testis

**Additional file 4.** List of downregulated genes related to spermatogenesis and sperm function in crossbred bulls

**Additional file 5.** Genes involved in WNT signaling in crossbred testis and their biological process (Elliptical) and pathway (hexagonal). Genes inside the box are upregulated and other genes are down regulated

**Additional file 6.** 10 highly upregulated and downregulated pathways in crossbred bull testis

**Additional file 7.** List of genes involved in upregulated and downregulated pathways related to spermatogenesis and sperm function in crossbred testis

**Additional file 8.** List of genes selected for real time expression analysis

**Additional file 9.** List of differentially expressed transcripts between Zebu and crossbred bull testis (1.5-fold change)

**Additional file 10.** Spermatogenesis and sperm function related up regulated genes in crossbred bull testis

## Data Availability

The raw data pertaining to the differentially expressed transcripts are given in Additional file 9. The datasets generated and/or analysed during the current study are available in the NCBI Gene Expression Omnibus repository (GSE153952, https://www.ncbi.nlm.nih.gov/geo/query/acc.cgi?acc=GSE153952).

## Abbreviations

PI4KB: Phosphatidylinositol 4-kinase beta
DPY19L2: Developmental Pluripotency-associated 19 like 2
RNA: Ribonucleic acid
mRNA: Messenger RNA
WNT: Wingless-related integration site
cAMP: Adenosine 3′,5′-cyclic monophosphate
*MSMB*: Microseminoprotein beta
EGF: Epidermal growth factor
*SPATA7*: Spermatogenesis associated 7
PANTHER: Protein Analysis Through Evolutionary Relationships
DAVID: Database for Annotation, Visualization and Integrated Discovery
PI3K-AKT: Phosphatidylinositol 3-kinase- protein kinase B
Jak-STAT: Janus kinases, and signal transducer and activator of transcription proteins
GABA: Gamma-Aminobutyric Acid
RT-qPCR: Real time quantitative polymerase chain reaction
PLCζ: Phospholipase C isoform zeta
cDNA: Complementary Deoxyribonucleic acid
cRNA: Complementary Ribonucleic acid
QC: Quality control
Cy3: Cyanine 3
PCR: Polymerase Chain Reaction
GAPDH: Glyceraldehyde 3-phosphate dehydrogenase
BACTIN: Beta actin
SPSS: Statistical Package for the Social Sciences
