# Supplementary material for: Sub-fertility in crossbred bulls: deciphering testicular level transcriptomic alterations between zebu (Bos indicus) and crossbred (Bos taurus x Bos indicus) bulls
Source: BMC Genomics. 2020 Jul 21;21:502. doi: 10.1186/s12864-020-06907-1 (PMC7372791; doi:10.1186/s12864-020-06907-1)
Supplement: Supplementary file 1 — Additional file 1. Top 10 abundant transcripts in bovine testis [file 12864_2020_6907_MOESM1_ESM.doc]

**Additional file 1: Top 10 most abundant transcripts in bovine testis**

| **Gene Symbol** | **Gene Name** | **Genbank Accession** |
| --- | --- | --- |
| *COX3* | Cytochrome c oxidase subunit III | U01924 |
| *TUBB2C* | Tubulin, beta 2C | NM_001034663 |
| *COX2* | Cytochrome c oxidase subunit II | DQ347621 |
| *PRM1* | Protamine 1 | NM_174156 |
| *TMSB4X* | Thymosin beta 4, X-linked | NM_001002885 |
| *FTH1* | Ferritin, heavy polypeptide 1 | NM_174062 |
| *ODF2* | Outer dense fiber of sperm tails 2 | NM_001038180 |
| *RPLP2* | Ribosomal protein, large, P2 | NM_174788 |
| *RPS10* | Ribosomal protein S10 | NM_001034716 |
| *RPS8* | Ribosomal protein S8 | NM_001025317 |
