# Supplementary figures and images for: Sub-fertility in crossbred bulls: deciphering testicular level transcriptomic alterations between zebu (Bos indicus) and crossbred (Bos taurus x Bos indicus) bulls
Source: BMC Genomics. 2020 Jul 21;21:502. doi: 10.1186/s12864-020-06907-1 (PMC7372791; doi:10.1186/s12864-020-06907-1)

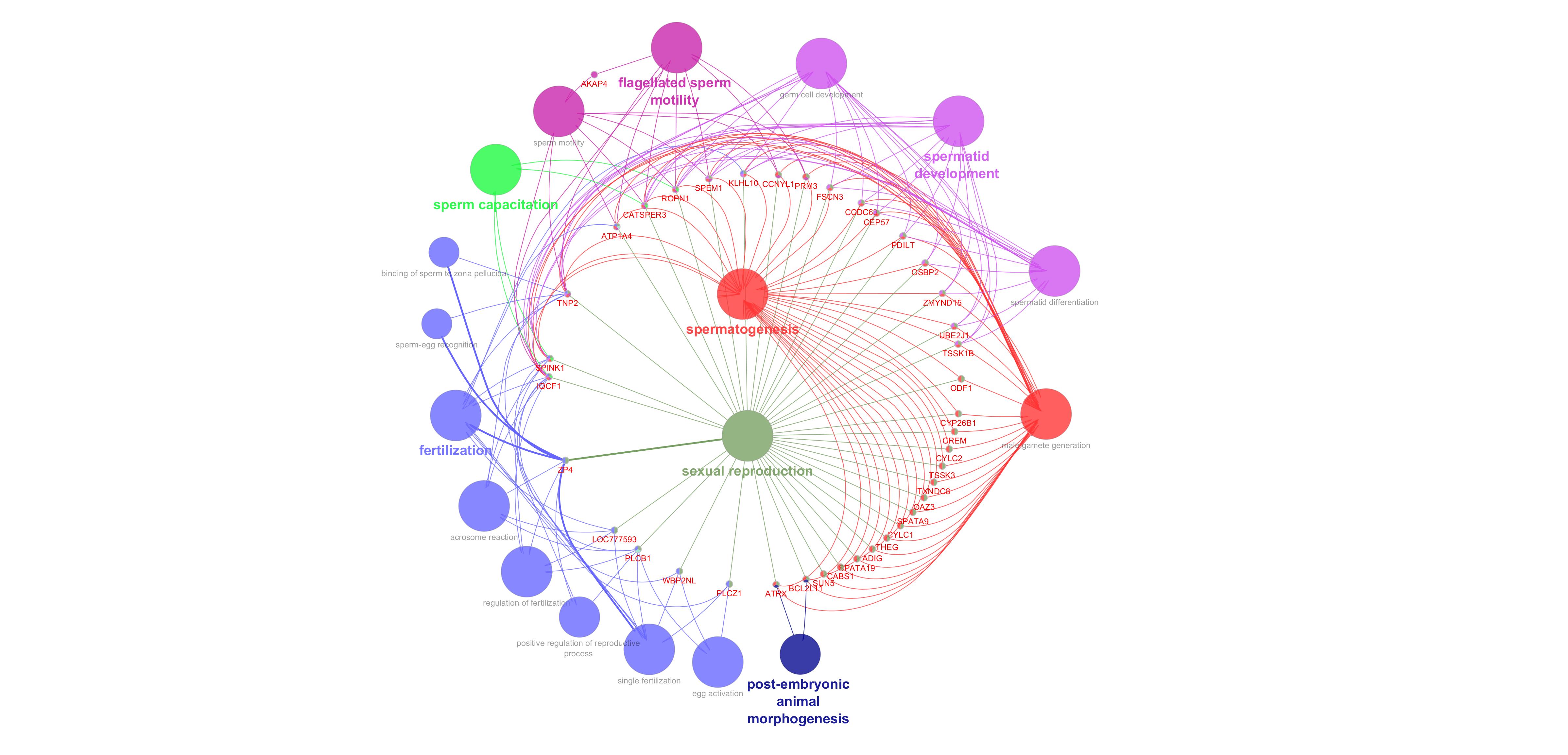

Supplement: Supplementary file 2 — Additional file 2. Share of biological process between upregulated genes related to spermatogenesis and sperm function in crossbred testis [file 12864_2020_6907_MOESM2_ESM.jpeg]

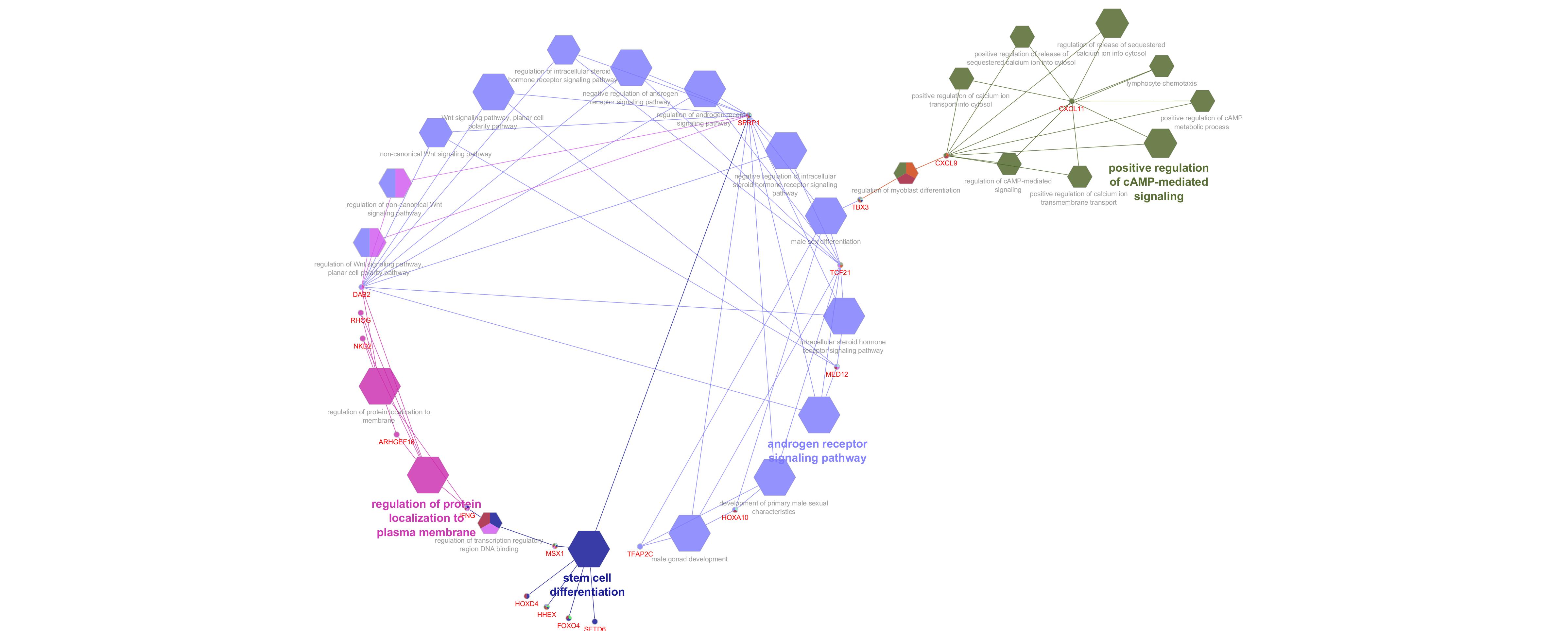

Supplement: Supplementary file 3 — Additional file 3. Share of biological process between downregulated genes related to spermatogenesis and sperm function in crossbred testis [file 12864_2020_6907_MOESM3_ESM.jpeg]

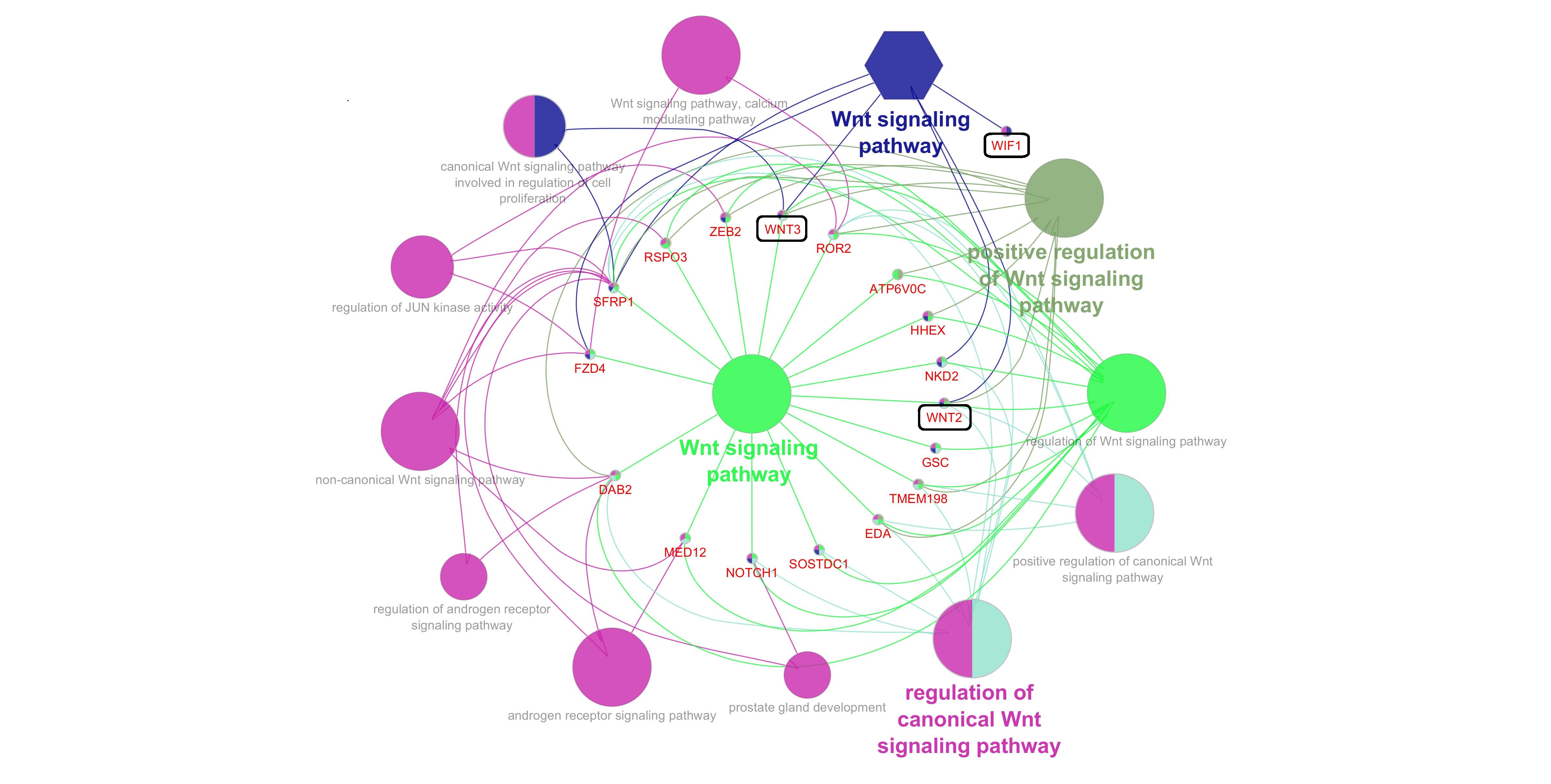

Supplement: Supplementary file 5 — Additional file 5. Genes involved in WNT signaling in crossbred testis and their biological process (Elliptical) and pathway (hexagonal). Genes inside the box are upregulated and other genes are down regulated [file 12864_2020_6907_MOESM5_ESM.jpeg]

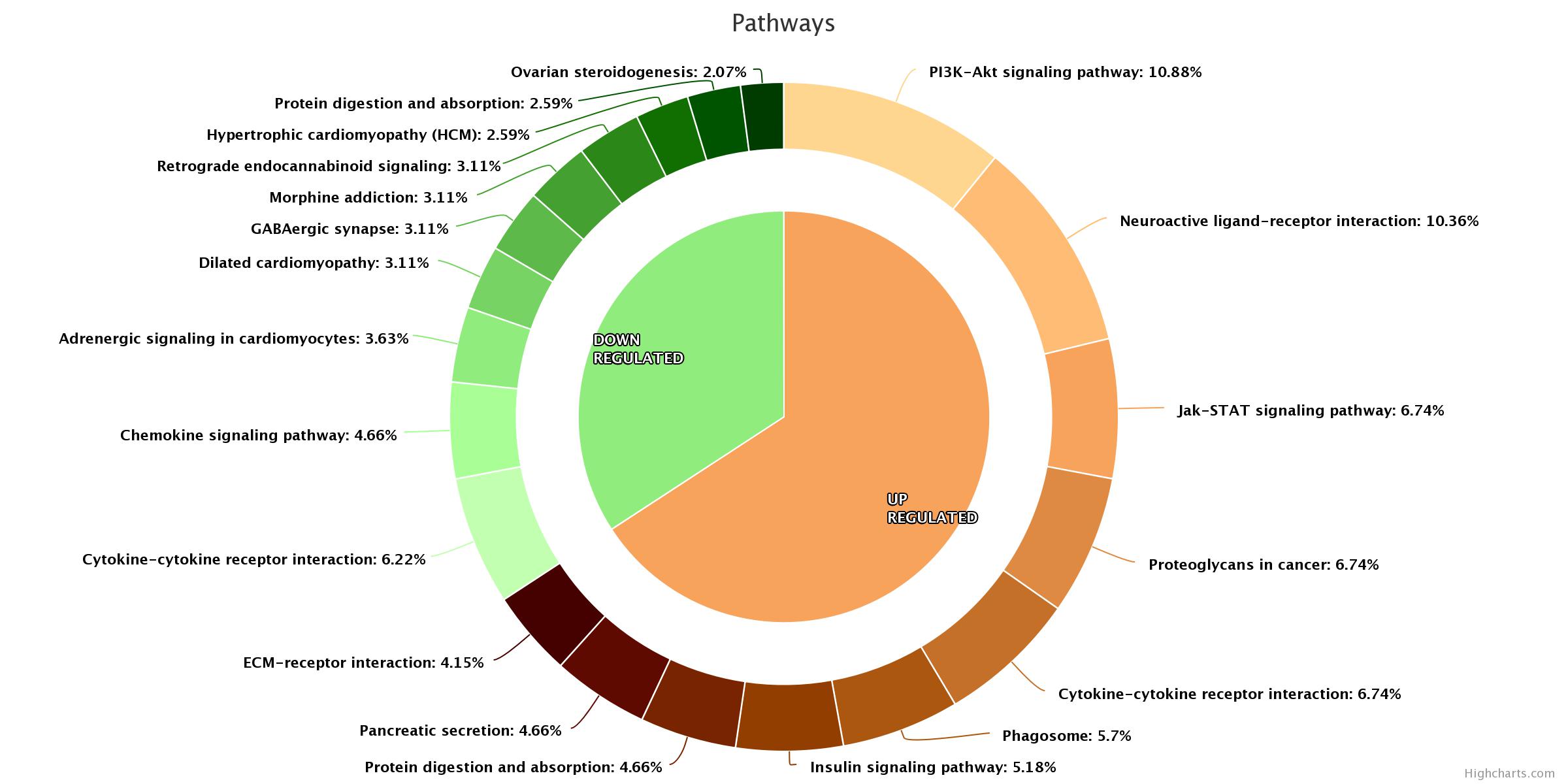

Supplement: Supplementary file 6 — Additional file 6. 10 highly upregulated and downregulated pathways in crossbred bull testis [file 12864_2020_6907_MOESM6_ESM.jpeg]
