# Supplementary material for: Sub-fertility in crossbred bulls: deciphering testicular level transcriptomic alterations between zebu (Bos indicus) and crossbred (Bos taurus x Bos indicus) bulls
Source: BMC Genomics. 2020 Jul 21;21:502. doi: 10.1186/s12864-020-06907-1 (PMC7372791; doi:10.1186/s12864-020-06907-1)
Supplement: Supplementary file 4 — Additional file 4. List of downregulated genes related to spermatogenesis and sperm function in crossbred bulls [file 12864_2020_6907_MOESM4_ESM.doc]

Additional file 4: List of downregulated genes related to spermatogenesis and sperm function in crossbred males

| **Function** | **P value** | **Count** | **Genes** |
| --- | --- | --- | --- |
| Biological Process | | | |
| Regulation of cell proliferation | 0.09 | 7 | *HHEX, DUSP15, CXCL9, NGFR, TFAP2C, CXCL11, TEC* |
| Regulation of canonical Wnt signaling pathway | 0.03 | 6 | *DAB2, RGS20, NOTCH1, NKD2, SOSTDC1, ROR2* |
| Stem cell differentiation | 0.01 | 4 | *MSX1, HOXD4, SETD6, FOXO4* |
| Positive regulation of establishment of protein localization to plasma membrane | 0.01 | 4 | *NKD2, IFNG, ARHGEF16, RHOG* |
| Stem cell population maintenance | 0.05 | 4 | *SALL4, TBX3, SETD6, MED12* |
| Male gonad development | 0.09 | 4 | *SFRP1, HOXA10, HOXA9, TFAP2C* |
| Single fertilization | 0.03 | 4 | *TRPC2, HOXA10, HOXA9, HOXD10* |
| Wnt signaling pathway, calcium modulating pathway | 0.06 | 2 | *ROR2, FZD4* |
| Positive regulation of cAMP metabolic process | 0.08 | 2 | *CXCL9, CXCL11* |
| Molecular Function | | | |
| Heparin binding | 0.08 | 6 | *FMOD, SFRP1, COL13A1, RSPO3, FGF1, CXCL11* |
| Wnt-activated receptor activity | 0.05 | 3 | *SFRP1, RYK, FZD4* |
