# Supplementary material for: Sub-fertility in crossbred bulls: deciphering testicular level transcriptomic alterations between zebu (Bos indicus) and crossbred (Bos taurus x Bos indicus) bulls
Source: BMC Genomics. 2020 Jul 21;21:502. doi: 10.1186/s12864-020-06907-1 (PMC7372791; doi:10.1186/s12864-020-06907-1)
Supplement: Supplementary file 7 — Additional file 7. List of genes involved in upregulated and downregulated pathways related to spermatogenesis and sperm function in crossbred testis [file 12864_2020_6907_MOESM7_ESM.doc]

Additional file 7: List of genes involved in upregulated and downregulated pathways related to spermatogenesis and sperm function in crossbred testis

| **Pathways** | **Count** | **P value** | **Genes** |
| --- | --- | --- | --- |
| Upregulated pathways | | | |
| PI3K-Akt signaling pathway | 21 | 0.02 | *YWHAZ, PIK3CD, GNG13, FASLG, VTN, ITGB3, GNG12, HGF, IRS1, RPTOR, BCL2L11, ITGAV, TNR, COMP, ITGB6, IFNB3, GYS2, RELN, COL24A1, PRL, LOC524810* |
| Jak-STAT signaling pathway | 13 | 0.008 | *IL5, CTF1, PIK3CD, SOCS7, IL21, IL12RB2, LEP, STAT4, IL20RB, IL20RA, IFNB3, PIAS2, PRL* |
| Downregulated pathways | | | |
| Retrograde endocannabinoid signaling | 5 | 0.05 | *GABRA2, GABRB3, ADCY9, MAPK13, GNB3* |
| GABAergic synapse | 5 | 0.03 | *GABRA2, SLC6A1, GABRB3, ADCY9, GNB3* |
| Ovarian steroidogenesis | 4 | 0.07 | *CYP17A1, HSD3B1, ADCY9, HSD17B7* |
