# Supplementary material for: Sub-fertility in crossbred bulls: deciphering testicular level transcriptomic alterations between zebu (Bos indicus) and crossbred (Bos taurus x Bos indicus) bulls
Source: BMC Genomics. 2020 Jul 21;21:502. doi: 10.1186/s12864-020-06907-1 (PMC7372791; doi:10.1186/s12864-020-06907-1)
Supplement: Supplementary file 8 — Additional file 8. List of genes selected for real time expression analysis [file 12864_2020_6907_MOESM8_ESM.doc]

Additional file 8: List of genes selected for real time expression analysis

| **Genes** | **Roles** | **Reference** | **Fold change in microarray** |
| --- | --- | --- | --- |
| *MSMB* | Inhibit acrosome reaction and sperm motility | Franchi *et al.,* 2008; Chao *et al.,* 1996 | 4.94 |
| *CCNYL1* | WNT signaling | Koch *et al.,* 2015 | 3.09 |
| *SPEM1* | Essential for spermatid maturation, but also involved in ubiquitination | Bao *et al.,* 2010 | 2.92 |
| *TNP2* | Histone to protamine transition | Oliva, 2006 | 1.74 |
| *TNP1* | Histone to protamine transition | Oliva, 2006 | 1.47 |
| *CRISP2* | Involved in sperm egg interaction and it’s released during acrosome reaction from acrosome | Nimlamool *et al*., 2013 | 1.41 |
| *PI4KB* | Sperm capacitation and acrosome reaction | Etkovitz *et al.,* 2007 | -9.20 |
| *DPY19L2* | Head elongation and acrosome formation | Koscinski *et al.,* 2011; Harbuz *et al.,* 2011 | -7.58 |
| *SPATA7* | Spermatogenesis | Zhang *et al.,* 2003 | -4.92 |
| *SOX2* | Early embryonic development | Campolo *et al*., 2013 | -1.23 |
