# Supplementary material for: Sub-fertility in crossbred bulls: deciphering testicular level transcriptomic alterations between zebu (Bos indicus) and crossbred (Bos taurus x Bos indicus) bulls
Source: BMC Genomics. 2020 Jul 21;21:502. doi: 10.1186/s12864-020-06907-1 (PMC7372791; doi:10.1186/s12864-020-06907-1)
Supplement: Supplementary file 10 — Additional file 10. Spermatogenesis and sperm function related up regulated genes in crossbred bull testis [file 12864_2020_6907_MOESM10_ESM.doc]

**Additional file 10: Spermatogenesis and sperm function related up regulated genes in crossbred bull testis**

| **Count** | **Genes** |
| --- | --- |
| 22 | **Spermatogenesis** |
|  | *CABS1:* calcium-binding protein, spermatid-specific 1*, CREM:* cAMP responsive element modulator*, SUN5:* Sad1 and UNC84 domain containing 5*, ATP1A4:* ATPase, Na+/K+ transporting, alpha 4 polypeptide*, THEG:* Theg homolog*, C8H9ORF24:* testes development-related NYD-SP22*, BCL2L11:* BCL2-like 11*, TNP2:* transition protein 2*, ATRX:* alpha thalassemia/mental retardation syndrome X-linked*, CYLC1:* cylicin, basic protein of sperm head cytoskeleton 1*, SPEM1:* spermatid maturation 1*, CYLC2:* cylicin, basic protein of sperm head cytoskeleton 2*, SPATA9:* spermatogenesis associated 9*, OAZ3:* ornithine decarboxylase antizyme 3*, PRM3*: protamine 3*, ADIG:* adipogenin*, TXNDC8:* thioredoxin domain containing 8*, SPATA19:* spermatogenesis associated 19*, CYP26B1:* cytochrome P450, family 26*,* subfamily B, polypeptide 1, *CCNYL1:* cyclin Y-like 1*, ODF1:* outer dense fiber of sperm tails 1*, TSSK3*: testis-specific serine kinase 3 |
| 22 | **Proteolysis** |
|  | *CELA3B:* chymotrypsin-like elastase family, member 3B*, PAG19:* pregnancy-associated glycoprotein 19*, PAG16:* pregnancy-associated glycoprotein 16*, PRSS58:* protease, serine, 58*, MGC157408:* pregnancy-associated glycoprotein*, PRSS1:* protease, serine, 1 (trypsin 1), *PAG12:* pregnancy-associated glycoprotein 12*, ANPEP:* alanyl (membrane) aminopeptidase*, PAG7:* pregnancy-associated glycoprotein 7*, PAG11:* pregnancy-associated glycoprotein 11*, ENPEP:* glutamyl aminopeptidase*, HGF:* hepatocyte growth factor, *MMP2:* matrix metallopeptidase 2*, MMP13:* matrix metallopeptidase 13*, LNPEP*: leucyl/cystinyl aminopeptidase*, CBLC:* Cas-Br-M (murine) ecotropic retroviral transforming sequence c*, DDI1:* DNA-damage inducible 1 homolog 1*, MGC157405: pregnancy-associated glycoprotein, RELN:* reelin*, CELA2A:* chymotrypsin-like elastase family*,* member 2A*, PAG4:* SMAD family member 3*, PRSS37:* protease, serine, 37 |
| 11 | **Ubiquitin protein ligase activity (Molecular function)** |
|  | *PLCZ1:* phospholipase C, zeta 1*, CBLC:* Cas-Br-M (murine) ecotropic retroviral transforming sequence c*, STAT4:* signal transducer and activator of transcription 4*, PLCE1: phospholipase C, epsilon 1, ATP2C1:* ATPase, Ca++ transporting, type 2C, member 1*, SLC35B2:* solute carrier family 35, member B2*, ZP4:* zona pellucida glycoprotein 4*, GNG13:* guanine nucleotide binding protein, gamma 13, *MAPKAPK2:* mitogen-activated protein kinase-activated protein kinase 2*, GNG12:* guanine nucleotide binding protein, gamma 12*, PLCB1:* phospholipase C*,* beta 1*, IRS1:* insulin receptor substrate 1 |
| 9 | **Spermatid development** |
|  | *CCDC63*: coiled-coil domain containing 63*, OSBP2:* oxysterol binding protein 2*, CEP57:* centrosomal protein 57kDa*, FSCN3*: fascin homolog 3, actin-bundling protein, testicular, *UBE2J1*: ubiquitin-conjugating enzyme E2*,* J1*, PDILT:* protein disulfide isomerase-like, testis expressed, *TSSK1B:* testis-specific serine kinase 1B*, KLHL10:* kelch-like 10*, ZMYND15:* zinc finger, MYND-type containing 15 |
| 7 | **Sperm motility** |
|  | *SPEM1:* spermatid maturation 1*, PRM3:* protamine 3*, CATSPER3:* cation channel, sperm associated 3, *CCNYL1*: cyclin Y-like 1*, ATP1A4*: ATPase, Na+/K+ transporting, alpha 4 polypeptide*, ROPN1:* rhophilin associated tail protein 1*, AKAP4:* A kinase (PRKA) anchor protein 4 |
| 4 | **Ubiquitin conjugating enzyme binding (Molecular function)** |
|  | *RNF144A*: ring finger protein 144A*, DCUN1D1:* defective in cullin neddylation 1, domain containing 1*, RNF138:* ring finger protein 138*, DCUN1D3*: defective in cullin neddylation 1, domain containing 3 |
| 3 | **Positive regulation of acrosome reaction** |
|  | *IQCF1:* IQ motif containing F1*, ZP4:* zona pellucida glycoprotein 4*, PLCB1:* phospholipase C, beta 1 |
| 2 | **Egg activation** |
|  | *PLCZ1:* phospholipase C, zeta 1*, WBP2NL:* WBP2 N-terminal like |
